# Microstructure and Properties of Surface Metal-Matrix Composite Reinforced with the Product of Vitrification of Asbestos-Cement Waste and CRT Glass Cullet

**DOI:** 10.3390/ma19101962

**Published:** 2026-05-10

**Authors:** Józef Iwaszko, Krzysztof Kudła, Małgorzata Lubas

**Affiliations:** 1 Department of Materials Engineering, Faculty of Production Engineering and Materials Technology, Czestochowa University of Technology (CUT), 19 Armii Krajowej Ave., 42-200 Czestochowa, Poland; malgorzata.lubas@pcz.pl; 2 Faculty of Mechanical Engineering, Czestochowa University of Technology (CUT), 21 Armii Krajowej Ave., 42-200 Czestochowa, Poland; krzysztof.kudla@pcz.pl

**Keywords:** friction stir processing, composite, vitrification, asbestos-cement waste, CRT glass cullet

## Abstract

The main aim of the work was to analyse the microstructure and selected properties of a metal-matrix surface composite reinforced with a product of vitrification of asbestos-cement waste (ACW) and glass cullet from cathode-ray tubes (CRTs). The composite matrix was an AA7075 (Al-5.5Zn-2.4Mg-1.6Cu-0.2Cr) aluminium alloy. The FSP (friction stir processing) method was used to produce the composite. The composites were tested in the context of the possibility of using vitrified material as a substitute for other reinforcing materials. As a result of treatment, a composite surface layer was obtained, characterised by uniform distribution of the reinforcing phase with a good bond with the matrix. This process was accompanied by strong grain refinement in the stirring zone and partial dissolution of intermetallic phases. These microstructural changes, combined with the introduction of hard particles into the metal-matrix, resulted in a significant increase in the composite’s hardness and wear resistance. As a result of the conducted research, it was found that using the product of vitrification of ACW and CRT cullet in the composites manufacturing process is beneficial, as it is not only a competitive solution to other reinforcing phases, but also an effective way to manage waste hazardous to the environment and humans, thus adding new functionalities to products processed in this way.

## 1. Introduction

The need to reduce the consumption of natural resources and the demand for their extraction forces science and industry to seek and implement new material solutions and manufacturing technologies that will have the least possible negative impact on the environment. Therefore, materials from disposal or recycling are increasingly being used in manufacturing processes. This action is consistent with the sustainable development strategy and demonstrates not only concern for the natural environment, but also for the economics of the project. Materials derived from disposal or recycling are increasingly being used, for example, in the production of modern engineering materials, including advanced composites. The reinforcing phase in such composites uses, for example, dust formed as a result of combustion in power plants, as well as ashes from the combustion of agricultural waste, e.g., rice husk ash, peanut shell ash, bamboo leaf ash [1]. For example, Juang and Li [2] used the stir casting method to produce an ADC10-2Mg aluminium alloy matrix composite reinforced with class F fly ash, in which the total content of SiO_2_ + Al_2_O_3_ + Fe_2_O_3_ exceeded 70%. The share of the reinforcing phase in the composites obtained in this way ranged from 3 to 15% by weight. The performed tests showed that the application of fly ash as a strengthening phase reduces the density of the aluminium composite and increases its hardness as well as tensile strength. However, as the amount of fly ash introduced into the composite matrix was increased, the authors observed a rise in porosity and a decrease in the plasticity of the composite. Fly ash was also employed by Shankar et al. [3]. In this case, the matrix was the A354 alloy (Al-Si-Mg-Cu). The composites were produced using the liquid metallurgy method. Composite rods containing 5, 10, and 15% by weight of fly ash were tested. The authors achieved the best results in the case of the composite containing the 5 wt% reinforcing phase, which they explain by a more uniform distribution of the fly ash particles and their perfect connection with the matrix material. The composite material containing 5% fly ash was characterised by higher hardness, tensile strength, and yield strength than the other composite samples. Bandhu et al. [4] utilised rice husk ash (RHA) particles as the reinforcing phase in aluminium metal-matrix composites. Composite samples were produced by stir casting and additionally subjected to accumulative roll bonding (ARB). Thanks to the use of a complex manufacturing technique, including classical casting, but also the ARB method, the uniform dispersion of rice husk ash particles within the metallic matrix and breaking of particle agglomerations were achieved, resulting in enhanced strength, tensile toughness, and wear resistance. In the authors’ opinion, rice husk ash particles are a cost-effective and applicable reinforcement in metal-matrix composites. Equally promising results were obtained by Kumar et al. [5], who used the stir casting process to produce a hybrid metal-matrix composite reinforced with industrial waste fly ash particles and B_4_C particles. The authors used different shares of reinforcing phases and found that the best effects are achieved when the total share of these phases in the composite is 10%, with equal shares of fly ash and boron carbide of 5%. With this combination of fly ash and B_4_C shares, the authors obtained 18.7% higher tensile strength, 11.3% higher hardness, and 38.6% higher compression strength than the unreinforced Al–Mg–Si-T6 heat-treated alloy. The composites containing 5% fly ash and 5% B_4_C reinforcing particles also showed less agglomeration and little particle clustering at grain boundaries.

Suganeswaran et al. [6] employed fly ash and emery-based particles. The composite matrix was the AA7075 aluminium alloy, and to produce the composite, the authors used the friction stirring processing method. The conducted tests revealed that the Fe_3_O_4_, Al_2_O_3_ and SiO_2_ present in the fly ash and emery, combined with the homogeneous dispersion of the reinforcing phase, contribute to the higher surface hardness of the 50E50FA hybrid composite sample. The composite samples were also characterised by higher impact strength and wear resistance. As the above literature review shows, waste materials have enormous application potential.

In this work, an innovative material obtained by simultaneous vitrification of ACW and cathode-ray tubes cullet was used as a reinforcing material in the metal-matrix composite. There is no information in the world literature regarding the use of vitrified materials in the production of composites. Our preliminary research, published in [7], indicated the possibility of using vitrified material in the manufacturing of composites via the modern FSP method. This study involved analysing the microstructure and selected properties of a composite reinforced with a vitrification product.

## 2. Materials and Research Methodology

The matrix of the composite material was AA7075 aluminium alloy in the precipitation hardened state (T6 state: solution hardened, guenched and artificially aged). AA7075 aluminium alloy is a high-quality material used in the automotive, military, shipbuilding, aerospace and construction industries, both in structural components (components of trucks, railway waggons, bridge load-bearing elements, highly stressed structural components) and for the manufacture of parts exposed to heavy wear, e.g., gears and shafts, worm gears, etc. [8]. The widespread use of this alloy is due to its excellent fatigue resistance and strength comparable to that of many structural steels, at a significantly lower weight [9].

**Figure 1:**
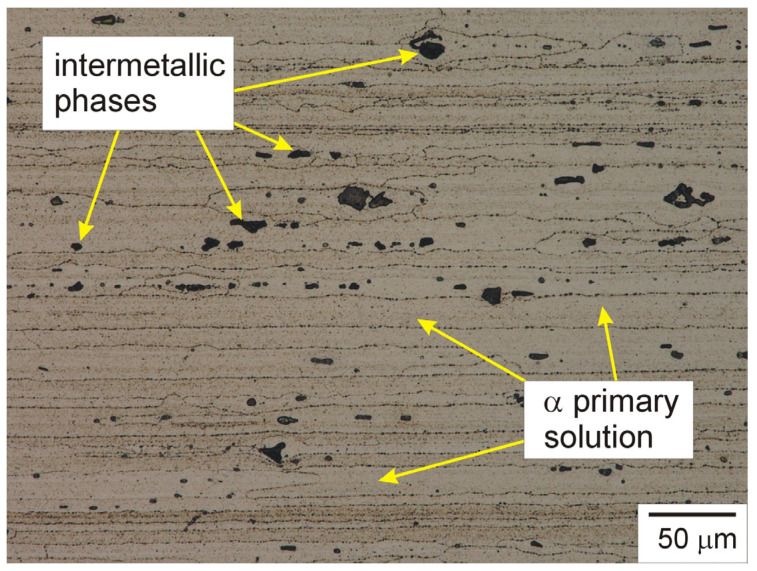
Microstructure of AA7075 aluminium alloy used to produce the composite, etched cross-section, light microscopy.

The main alloying elements in AA7075 aluminium alloy are zinc, magnesium, and copper. The detailed chemical composition of the alloy is presented in Table 1, and an example microstructure of the alloy is shown in Figure 1.

**Figure 2:**
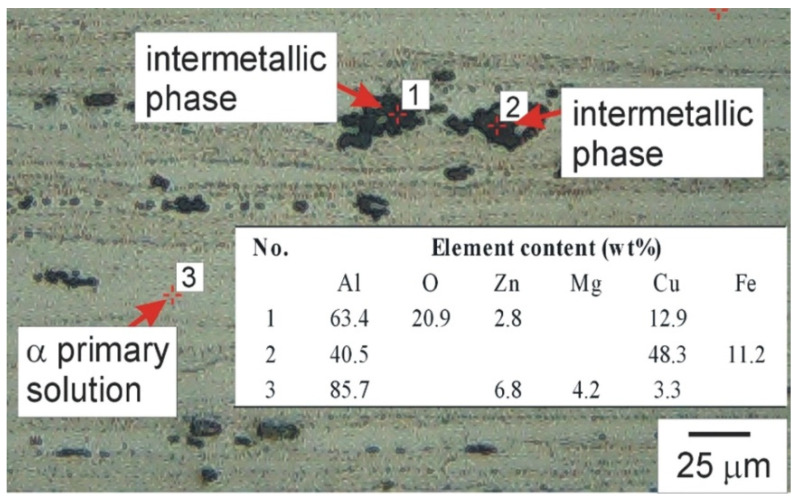
Results of chemical composition of intermetallic phases and α primary solution, etched cross-section, light microscopy with EA-300 laser elemental analysis head.

Microscopic examination of the AA7075 alloy performed using a Keyence_VHX-7000 light microscope (Keyence Ltd., Osaka, Japan) revealed the presence of highly elongated and directed grains of the α primary solution, the characteristic shape of which was a consequence of the rolling process. The grain sizes ranged from 80 µm to even 700 µm, with the average grain length being approximately 290 ± 10 µm. The alloy contained numerous precipitates of intermetallic phases, the presence of which was a consequence of both the solidification process and heat treatment. These phases were characterised by various chemical compositions and morphologies. Two complementary research methods were used to determine the chemical composition of intermetallic phases: Laser-Induced Breakdown Spectroscopy (LIBS) and EDS. For LIBS, an EA-300 laser elemental analysis head (Keyence Ltd., Mechelen, Belgium) coupled to a Keyence_VHX digital microscope was used. EDS analysis was performed using an Oxford Instruments microanalyser (Oxford_Instruments Plc., Abingdon, UK) coupled to a JOEL scanning electron microscope. The conducted studies revealed the dominance of precipitates containing aluminium, zinc, and copper, but also the presence of precipitates rich in aluminium, copper, and iron, as well as less numerous phases rich in aluminium, zinc, and magnesium, and phases containing aluminium, silicon, and zinc. Example results of the chemical composition of intermetallic phases and the α primary solution determined using the LIBS method are shown in Figure 2.

The precipitates occurred both on the grain boundaries of the α primary solution, but it is worth noting that the precipitates occurring inside these grains were also no less numerous, which is clearly visible in Figure 1. Intermetallic phases affect a number of properties of the aluminium alloy, including such key properties as mechanical properties and corrosion susceptibility; hence, the changes these phases undergo should always constitute a subject of analysis in assessing the hardness and wear resistance of the AA7075 alloy. Such an assessment was also carried out as part of this work.

**Figure 3:**
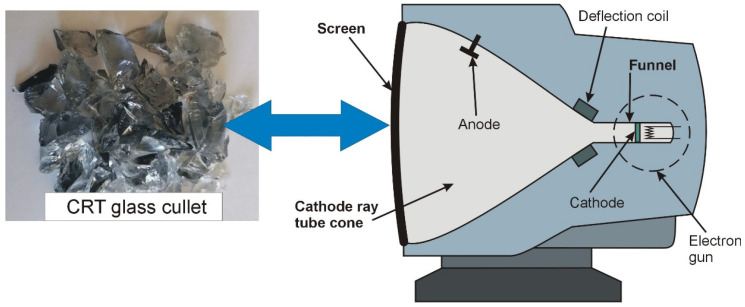
Construction diagram of CRT.

The reinforcing phase in the designed composite was the vitrification product of ACW (eternity) and CRT glass cullet obtained from the front screen part of the picture tube (Figure 3). The chemical composition studies were performed using an Axios Max WD-XRF X-ray fluorescence spectrometer (Malvern Panalytical Ltd., Malvern, UK). The results of the ACW and CRT cullet analysis are presented in Table 2 and Table 3.

The chemical composition of a material is crucial to the vitrification process, influencing the material’s ability to form a glassy state, the glass transition temperature, the material’s viscosity, and the kinetics of crystallisation. Materials containing network-forming components (e.g., SiO_2_, B_2_O_3_) undergo vitrification more readily. These components hinder the crystallisation process by stabilising the amorphous structure. Silica, which in this study was sourced from both asbestos waste and CRT cullet, increases the material’s viscosity, hindering the movement of atoms and the formation of an ordered crystal structure. Alkali metal oxides Na_2_O and K_2_O, sourced from cullet in this study, act as crystal modifiers, lowering the melting point, glass transition temperature, and viscosity of the material, allowing vitrification at lower temperatures. However, these oxides increase the material’s susceptibility to crystallisation. The course of the vitrification process, the required cooling rate, and the glass transition temperature, therefore, depend significantly on the chemical composition of the components undergoing vitrification.

Both the CRT glass cullet and ACW were ground in a FRITSCH_Pulverisette 6 planetary mill (FRITSCH GmbH, Idar-Oberstein, Germany). The grinding process was carried out dry, using a grinding bowl with a capacity of 250 cm^3^ and a grinder in the form of balls with a diameter of 18 mm. Both the grinding bowl and the grinding elements are made of zirconium oxide. The grinding time was 5 min, and the rotation speed was 300 rpm. The ground ACW and glass cullet were mixed in a 50:50 ratio and then melted in a furnace at a temperature of 1400 °C for 90 min. Cooling of the vitrified material was performed in a manner analogous to traditional glass production. The vitrification effect was achieved by pouring the molten material onto a cold steel plate and cooling the melt from 1400 °C to ambient temperature. The cooling method of the molten material and the resulting cooling intensity are, along with the chemical composition, equally important parameters influencing the course and final effect of the vitrification process. Vitrification requires that the cooling intensity be sufficiently high to prevent diffusional processes of nucleation and nucleus growth, leading to the formation of a crystalline structure. The research results presented in [7] by the authors of this study demonstrated that the cooling method used in the vitrification process is sufficient to effectively block crystallisation. Studies conducted using X-ray structural analysis revealed the formation of a fully amorphous structure, as evidenced by the absence of crystalline peaks in the diffraction pattern and the presence of a characteristic broad amorphous halo.

**Figure 4:**
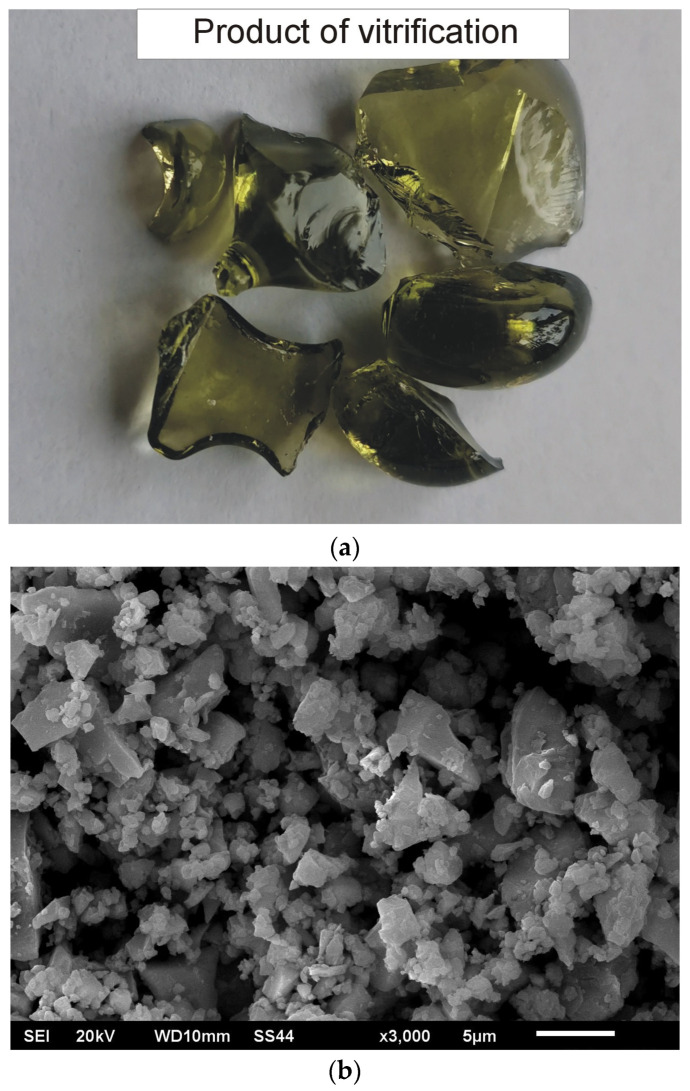
Vitrification product of ACW and CRT cullet (**a**), ground vitrified material (**b**). SEM (**b**).

The vitrification product had the form of a solid, transparent block with a greenish colour and a small number of bubbles (Figure 4a). No phases with fibrous morphology were found in the material, which proves that, as a result of vitrification, respirable asbestos fibres, the presence of which in the asbestos was revealed by microstructural tests, were completely eliminated during processing. Respirable asbestos fibres, i.e., less than 3 μm in diameter and more than 5 μm long, can cause serious respiratory diseases, including asbestosis and lung cancer, and are the main factor behind the pathogenic properties of this material.

The chemical composition tests performed using the WD-XRF method showed in the vitrified material structure the presence of both components originating from the ACW and the CRT cullet, including heavy metals Ba and Sr. These metals were added in the form of oxides to the cathode ray tube glass [10]. The chemical composition of the vitrified material is presented in Table 4.

Hardness measurements were carried out using a Shimatzu_HMV-G20 microhardness tester (Shimatzu Corp., Kyoto, Japan) with a Vickers penetrator. For vitrified material and glass cullet, the loading time was 10 s, and the load was 1.961 N. In the case of composites, a load of 980.7 mN and a loading time of 10 s were applied, and the hardness was measured on the cross-sections of the samples. The average distance between the Vickers hardness indentations was approximately 160 μm, whereas in the surface zone, approximately 1 mm thick, measurements were performed more densely, with a distance of approximately 100 μm between indentations.

The loading time and the applied load in hardness measurements should take into account the properties of the tested material, particularly the variation in plastic deformation of the material depending on the time the indenter is subjected to the specified force. The measurement is most often performed using a time of 10 to 15 s. A shorter time is recommended for materials that, under measurement conditions, exhibit plastic deformation independent of the loading time, while longer times are used for materials that, as a result of the measurement, exhibit a significant dependence of deformations on the indenter loading time. Due to the fact that the hardness tests were performed on, among others, CRT glass and vitrified material, in the case of which the application of a higher load could lead to the formation of microcracks in the corners of the indentation, it was decided to use a load of 10 s, at which such an effect was not observed during previous hardness measurements.

The tests showed that the vitrified material had a higher hardness than cathode ray tube glass, which was a key component in its production process. The CRT glass had a hardness of 596 HV0.2, while the vitrified product had a hardness of 637 HV0.2. The differences in the hardness of vitrified material and CRT glass resulted primarily from differences in their chemical compositions. The vitrification product featured a higher content of oxides such as Al_2_O_3_, ZrO_2_, MgO, and CaO, which positively affect the hardness of glass. On the other hand, a significantly lower content of alkali oxides Na_2_O and K_2_O was found, which in turn weakens the hardness of glass [11].

Tribological tests of the composites were performed using a T_01M pin-on-disc tester (ITEE, Radom, Poland) under dry friction conditions. The samples were in the form of a pin with a 4 mm diameter. A test was conducted with a load of 20 N and a disc rotational speed of 150 rpm. Total wear distance was 1500 m. The measure of wear was the linear change in the length of the tested sample as a function of time.

The vitrified material was ground using a planetary mill. The rotational speed of the grinding bowl was 300 rpm, and the grinding time was 5 min. The obtained powder was subjected to microscopic examination to assess the geometric structure of the surface and the shape of the obtained particles. Microstructural studies of the powdered vitrification product were performed using a JEOL_JSM-6610LV scanning electron microscope (JEOL Ltd., Tokyo, Japan). SEM studies showed that the vitrified particles are characterised by a polyhedral shape, a low degree of surface development, and the presence of distinct cleavage planes typical of glass (Figure 4b). The particle sizes ranged from tenths of a micrometre to approximately 16 micrometres, with the dominant fraction being particles ranging from 0.5 to 2.5 micrometres. The material prepared in this way was used as a reinforcing phase in the composite.

Technical SiC powder, which was used to produce the reference composite, was also subjected to SEM tests. Silicon carbide is a good reference material because it is one of the most commonly employed phases to strengthen composites. This is because it is a material characterised not only by high hardness and wear resistance, but also by high strength, low thermal expansion, with equally high chemical resistance, including resistance to acids and alkalis [12]. The SiC powder used in this work was characterised by a polyhedral shape and a low specific surface area. The SiC particle dimensions ranged from 1 to 25 micrometres, with the dominant fraction being particles with dimensions from 2 to 7 micrometres.

## 3. Composite Production

The FSP method was used to produce the composite. This method, similarly to the FSW (friction stir welding) method from which FSP is derived, uses a cylindrical tool that is set in rotation during machining [13]. The tool is used to plasticise the matrix material and mix the composite components [14,15]. Plasticisation of the material occurs due to the heat generated by the friction between the tool and the sample surface and the strong plastic deformation of the material. The amount of heat generated during friction stir processing results from the machining parameters used, but also from the tool geometry [16,17,18]. It is worth noting that both the shoulder and the tool pin contribute to the thermal effect, but the pin’s contribution to this process is smaller, and furthermore, greater temperature fluctuations may occur where the pin interacts with the material.

**Figure 5:**
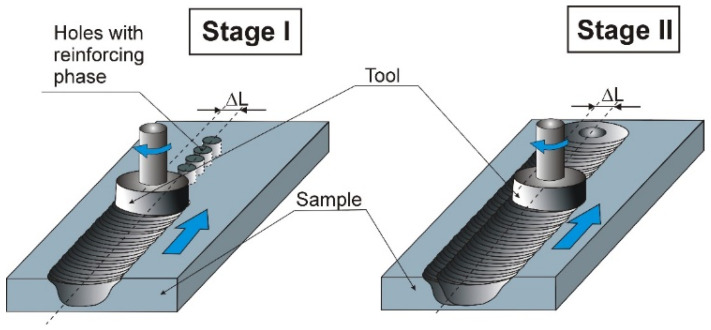
Diagram of FSP hole method with shifted working zone of the tool.

The FSP method uses various methods for introducing the reinforcing material into the composite matrix. The most popular method is the groove method, but an alternative solution is the hole method [19,20]. In the case of a hole variant of FSP, the reinforcing phase is placed in separate holes drilled in the surface of the material constituting the matrix of the composite. One variation in the hole method is the hole solution with a shifted working zone for the pin [21]. This solution consists of two successive stages. In the first stage, the rotating tool moves at a certain distance ΔL from the line defined by the holes drilled in the sample surface, and in the second stage, the tool moves along the original line of holes. This solution allows for more uniform distribution of the reinforcing phase in the matrix and reduces tool wear by limiting direct contact between the hard reinforcing phase and the tool as the pin penetrates the material. Based on previous studies and the resulting practical conclusions, a ΔL value of 2.5 mm was assumed for the purposes of this treatment. A diagram of the FSP method with a shifted pin working zone is presented in Figure 5.

The advantage of FSP in the production of composites over competitive casting technologies is that no liquid phase is formed during processing, which eliminates the potential problem of a lack of wettability or insufficient wettability of the particle surface by the liquid matrix. This, in turn, affects the quality of particle bonding with the matrix.

FSP was performed using a 3-axis CNC milling machine. The tool was made of X37CrMoV5-1 tool steel. The tool pin was conical, had a threaded side surface, and was 5 mm long. The tool shoulder diameter was 18 mm. The reinforcing material was placed in holes 2 mm in diameter and 4.6 mm deep. Friction stir processing of SiC particle-reinforced materials and vitrified particle-reinforced compositions was performed with identical tool rotational speeds and feed rates of 400 rpm and 30 mm/min, respectively. Temperature control in the friction-stir-processed material and, consequently, changes in its microstructure were achieved by selecting key process parameters: tool rotational speed and feed rate. Increasing the rotational speed increases the amount of heat generated in the working zone and raises the peak temperature in the modified material. Increasing the feed rate, in turn, shortens the tool-material interaction time and the friction time, thus lowering the peak temperature to which the material is heated during friction stir processing. Excessively high temperatures generated in the material during processing are unfavourable because they reduce grain refinement. Therefore, the selection of processing parameters must be based on a rational assessment of the material’s degree of plasticisation and the microstructural effects of treatment. The selection of the key FSP parameters in this work was based on previous research and tests optimising the parameters for manufacturing composites based on the AA7075 aluminium alloy.

**Figure 6:**
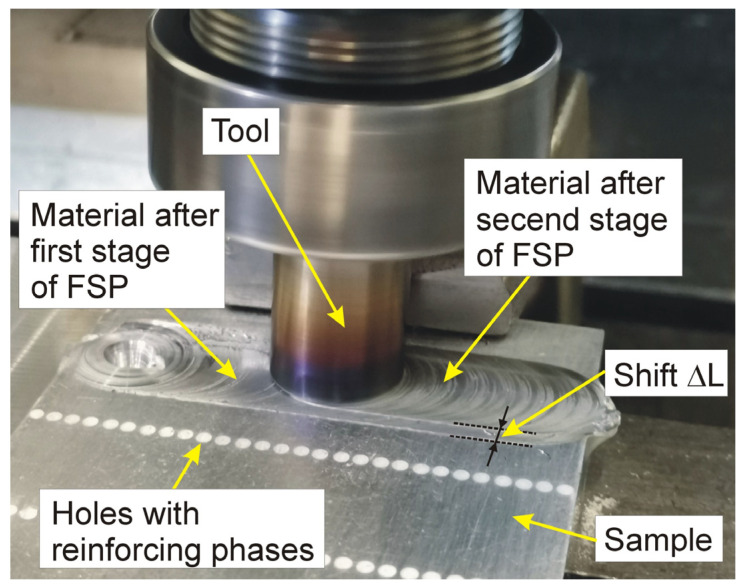
Composite layer production station using FSP method.

The composite layer production station using the FSP method is shown in Figure 6.

## 4. Results and Discussion

### 4.1. Microstructural Studies

**Figure 7:**
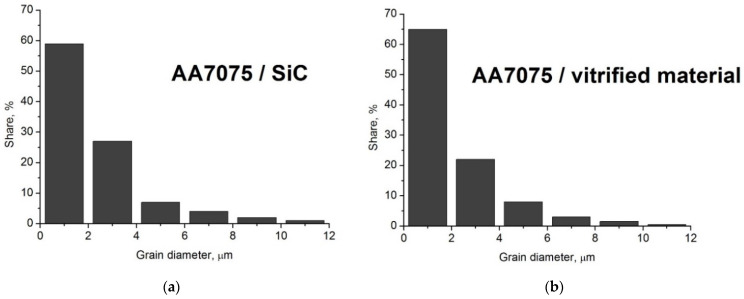
Histograms of grain sizes in AA7075/SiC composite (**a**), in AA7075/vitrified material composite (**b**).

The friction-stir-processed samples were subjected to comparative microstructural studies to assess the size and extent of changes induced by the treatment. In these studies, attention was primarily paid to the uniformity of the reinforcing phase distribution in the matrix, the quality of the bond between the reinforcing phase particles and the matrix, and microstructural changes in the composite matrix, i.e., features of the composite that indicate correct manufacture and application potential. Both the composite reinforced with the vitrification product and the reference composite reinforced with silicon carbide particles were tested. Specimens were cut from the samples across the friction-stir-processed zone and also from the base materials. The tests carried out showed that a composite microstructure was formed in the surface layer of the AA7075 alloy, featuring uniformity in the distribution of reinforcing phase particles in the matrix volume. It was also found that the microstructural changes that occurred in the composite matrix were analogous to those observed in metallic materials subjected to FSP treatment, namely strong grain refinement combined with the formation of characteristic zones, i.e., clearly dominant the stirring zone (SZ), the thermomechanically affected zone (TMAZ) and the heat affected zone (HAZ) [22]. The SZ was located in the central part of the friction-stir-processed zone, while the thermomechanically affected zone was located between the stirring zone and the heat-affected zone. The thickness of the composite surface layer was approximately 5 mm, which roughly corresponded to the depth the working tool pin penetrated the material. The matrix grain size in the stirred zone was significantly reduced by a factor of nearly 150 compared to the initial state. The average grain size in the stirred zone was approximately 2 ± 0.5 µm, while the average grain size before treatment was nearly 290 ± 10 μm. A similar degree of grain refinement was also found in the case of the composite reinforced with silicon carbide particles. Example histograms of grain sizes in the AA7075/SiC composite and the AA7075/vitrified material composite are shown in Figure 7.

The degree of grain refinement is, among other things, the result of the amount of plastic deformation of the material, the peak temperature generated in the sample, and the time of heat exposure to the material [23]. The degree of grain refinement is also influenced by the so-called Zener effect (Zener pinning) [24]. This effect involves the localization of finely dispersed particles at the grain boundaries, which makes the movement of recrystallization boundaries/fronts much more difficult or even impossible. It is worth noting that in the case of vitrified material, particles with dimensions not exceeding a few micrometres dominated, hence the grain growth must have been inhibited as a consequence of the Zener effect. Grain refinement is most beneficial because it usually leads to improvement of the material’s mechanical properties, as well as wear resistance, and is one of the main goals of performing FSP. The matrix grain refinement was also observed in the TMAZ, but the grains in this zone had a less regular shape than the grains in the SZ. This was due to the fact that in the TMAZ, dynamic recrystallization does not occur or occurs to a limited extent. The temperature in the TMAZ is usually lower than in the stirring zone, ranging from 0.6 to 0.7 of the material’s melting point [25,26,27,28,29]. The highest temperature during FSP occurs in the stirring zone, and the material in this zone is also subjected to the strongest plastic deformation. The conditions in which the surface layer is formed determine the size and shape of the grain.

Microscopic examinations revealed that the surface treatment led to partial dissolution of intermetallic phases in the aluminium alloy, and those that were not dissolved were redistributed during FSP, which resulted in their more uniform dispersion in the matrix. Research did not demonstrate any change in their original shape and size. Intermetallic phases have a significant impact on the properties of the aluminium alloy, especially its mechanical and tribological properties. Therefore, when analysing the results of hardness and tribological wear measurements, the influence of intermetallic phases on these composite properties was taken into account.

**Figure 8:**
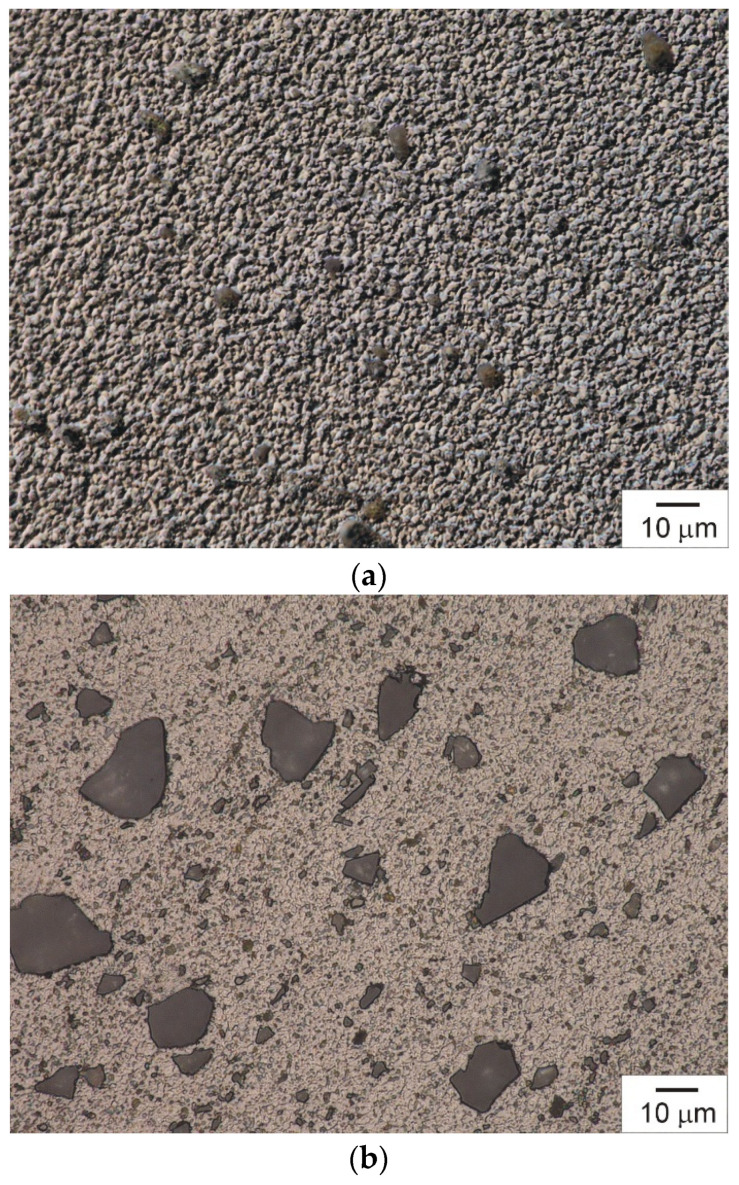

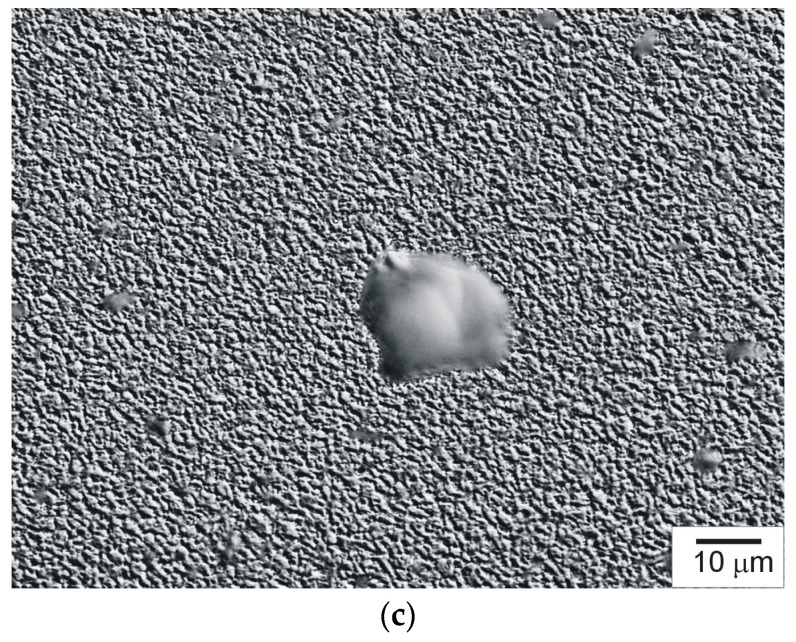
Vitrification product-reinforced composite (**a**), SiC particle-reinforced composite (**b**), matrix-reinforcement phase interface (**c**). Light microscopy. Optical shadow effect mode (**a**,**c**).

One of the key microstructural parameters determining the quality of the composite and its application potential is the distribution of reinforcing phase particles in the material and the presence of agglomerates in the microstructure. The presence of agglomerates or an uneven particle distribution in the composite matrix adversely affects the composite’s mechanical properties, indicating an insufficient mixing process. In the case of the composites analysed in this work, tests showed that the particles of the reinforcing phase in the produced composites were mostly evenly distributed throughout the matrix (Figure 8a,b) and well bonded to the composite matrix (Figure 8c). It is worth noting that the uniformity of the distribution of reinforcing phase particles through the matrix is influenced not only by the processing parameters but also by the shape of the particles. Particles with a more complex shape and a large specific surface area have a greater tendency to form clusters and jam each other. The particles of the vitrification product and silicon carbide used in this work featured a characteristically polyhedral shape and a low-developed geometric surface structure, which undoubtedly facilitated the uniform distribution of the reinforcing phase in the plasticised matrix. However, comparative studies of the microstructure of the composite reinforced with a vitrification product (Figure 8a) and the microstructure of the composite reinforced with silicon carbide particles (Figure 8b) revealed a stronger uniformity of the reinforcing phase distribution in the AA7075/SiC composite.

### 4.2. Measurement of Composite Hardness and Wear Resistance

Hardness and wear resistance are the basic parameters determining the application value of the composite, and they also constitute an objective assessment of the effectiveness of the given processing and the suitability of the applied reinforcing materials. Therefore, as part of this work, in addition to microstructural tests describing the changes induced in the surface layer of the AA7075 alloy, measurements of the hardness and wear resistance of the aluminium alloy before and after processing were performed.

Hardness measurements showed that as a result of friction stir processing and the introduction of hard vitrified material particles into the AA7075 alloy matrix, the material became approximately 39% harder. The composite hardness was 135 HV0.1, while the hardness of the AA7075 alloy was 97 HV0.1. However, compared to the reference sample reinforced with silicon carbide particles, a slightly lower hardness was found (the increase in hardness in the case of the AA7075/SiC composite was approximately 43%) and a larger amplitude of hardness value dispersion.

**Figure 9:**
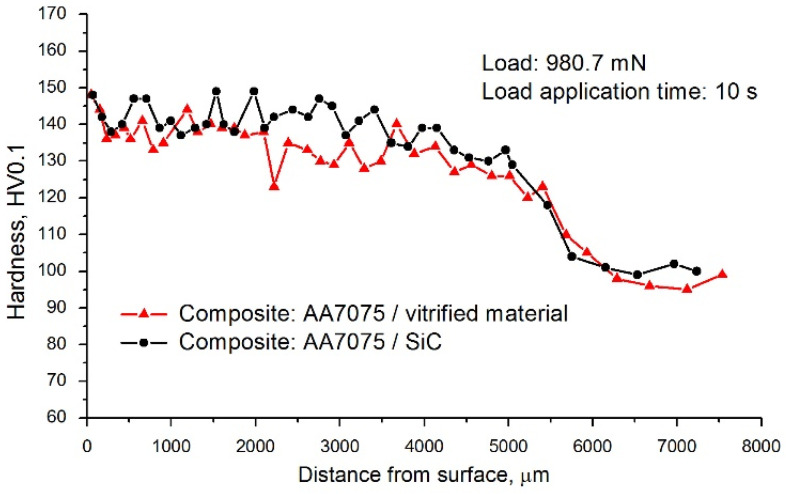
Results of hardness measurement of composite samples.

The less uniform hardness distribution in the case of particles of vitrified material can be explained by the more complex particle morphology, the larger specific surface area of the particles, and their smaller dimensions. These factors varied the way and how easily particles moved through the plasticised matrix, resulting in a less uniform dispersion of the vitrified material in the composite matrix. Nevertheless, when comparing the hardness of the composite reinforced with silicon carbide particles and the vitrified material, one, it should be emphasised that these hardness results were much higher than the hardness of the starting material. Graphs illustrating changes in material hardness as a function of distance from the surface are shown in Figure 9.

Analysing the microhardness changes as a function of distance from the surface, it can be seen that a significant decrease in hardness occurs only at a distance of approximately 5 mm from the sample surface. This is due to the range of microstructural changes induced by friction stir processing. This range generally corresponds to the pin’s dimensions and the tool’s effective penetration depth into the composite material. It is worth noting that the hardness values within a distance of up to 5 mm from the surface exhibit only slight fluctuations, indicating the material’s microstructural homogeneity.

**Figure 10:**
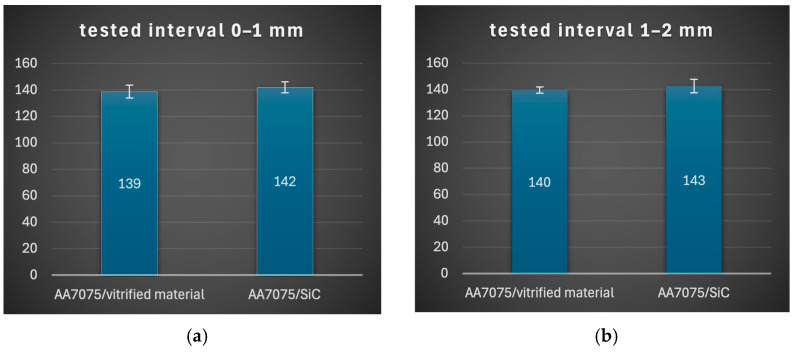

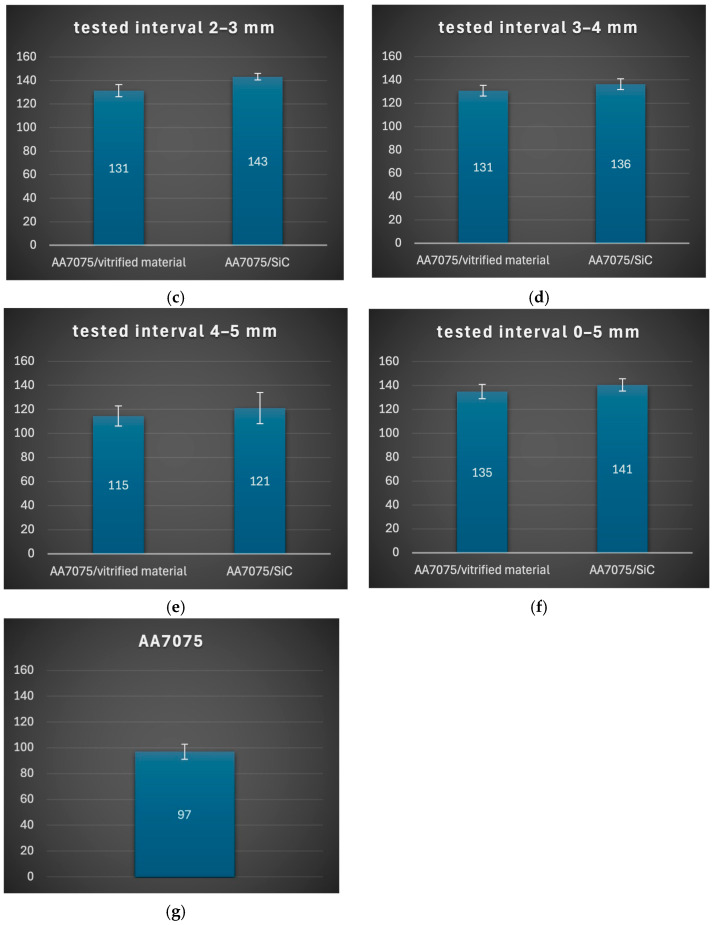
Graphs of mean hardness values and confidence intervals as a function of distance from surface: (**a**) 0–1 mm, (**b**) 1–2 mm, (**c**) 2–3 mm, (**d**) 3–4 mm, (**e**) 4–5 mm, (**f**) 0–5 mm, (**g**) for AA7075 alloy (>6 mm).

The errors in the hardness data are shown in Figure 10. The mean hardness values as a function of distance from the surface, along with the confidence intervals, are shown in Table 5.

According to the Hall-Petch rule, the hardness of a material is inversely proportional to its grain size [30,31]. This means that the greater the grain refinement, the harder the material. As grain size decreases, the total number of grain boundaries, which act as a barrier to dislocations and cause their immobilisation, increases. This phenomenon is known as grain boundary strengthening, or Hall-Petch strengthening [32,33]. It is worth noting, however, that a material’s hardness is not solely determined by grain size; there are many other factors that the Hall-Petch rule unfortunately does not account for. In the case of aluminium alloys, intermetallic phases have an equally significant impact on their hardness. As a result of FSP treatment, some of these precipitates were dissolved, which could result in a decrease in hardness. Nonetheless, previous research by the authors of this work showed that the change in hardness caused by the dissolution of intermetallic phases can be effectively compensated by strong grain refinement caused by dynamic recrystallization of the material [34]. In this case, the introduction of a reinforcing phase into the hard matrix is an additional factor stimulating growth in the hardness of the composite. The rise in hardness observed in this study is therefore a consequence of the introduction of hard particles into the aluminium matrix, but also of solid-state transformations caused by FSP, resulting in strong grain refinement.

**Figure 11:**
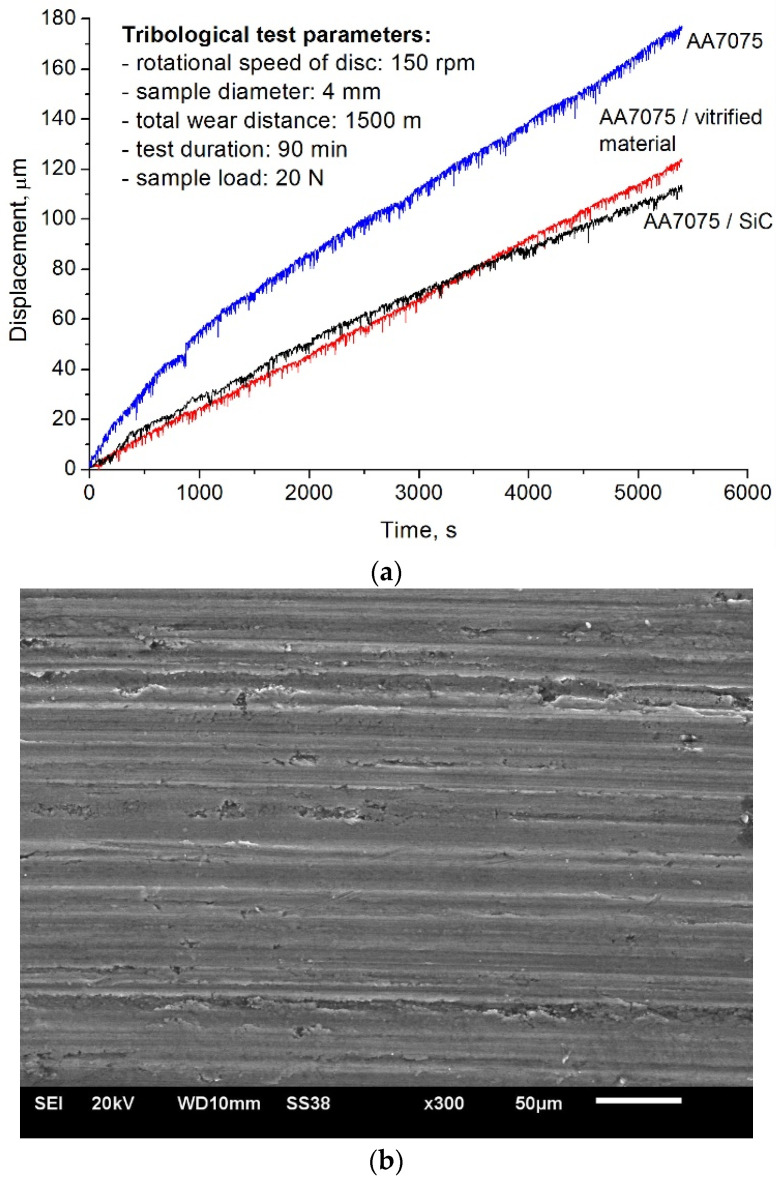
Tribological test results of composite samples and AA7075 alloy (**a**), surface morphology of composite reinforced with vitrified material particles (**b**).

Based on the hardness measurement results, the composite is expected to exhibit higher wear resistance than the starting AA7075 alloy. According to Archard’s equation, the wear intensity of a material is inversely proportional to the hardness of the material [35]; the higher the hardness, the greater the wear resistance. The tribological test results presented in Figure 11a clearly confirm the Archard equation. The tribological tests showed a 30.4% lower loss of the tested sample compared to its counterpart without the reinforcing phase. The lowest linear sample wear was observed for the sample reinforced with silicon carbide particles, which is undoubtedly due to more favourable microstructural changes in this composite, especially the more uniform dispersion of the reinforcing phase in the matrix. Patil et al. [36] also found that the wear rate of composites increases when agglomerates of particles are present in the microstructure. The authors suggest that when the reinforcing phase particles are uniformly distributed in the composite matrix, the particles act as optimised load-bearing asperities. The presence of agglomerates, in turn, contributes to the chipping of reinforcing phase particles from the composite matrix due to insufficient interfacial bonding between the agglomerates and the composite matrix.

It is worth noting, however, that the differences in the wear resistance of the composite samples reinforced with silicon carbide particles and the vitrified material were not significant, and what is more, until about the 60th minute of the test, the wear intensity of the sample reinforced with the vitrified material was even lower, as can be seen from the curves presented in Figure 11a. Only in the later phase of the test did the advantage of the material reinforced with silicon carbide particles become visible. The uniformity of particle distribution depends on the physicochemical properties of the powder, especially the dimensions, the shape of the particles, and the geometric structure of their surface, but also on the degree of plasticisation of the matrix material and the intensity of mixing during processing. According to the authors, these factors determined the higher wear resistance of the composite reinforced with silicon carbide particles.

Examination by scanning microscope of the sample surfaces after tribological testing revealed the presence of parallel cracks in the geometric structure, resulting from micro-ploughing and micro-cutting (Figure 11b). This was the dominant wear effect on the samples, proving that an abrasive wear mechanism occurred during the tribological test. The hard particles of the reinforcing phase are many times harder than the aluminium matrix; therefore, during tribological testing, it is mainly the particles that transfer the load. Moreover, they reduce the contact surface of the sample with the counter-sample, which reduces or even eliminates the share of adhesive wear in favour of abrasive wear.

## 5. Summary

**Figure 12:**
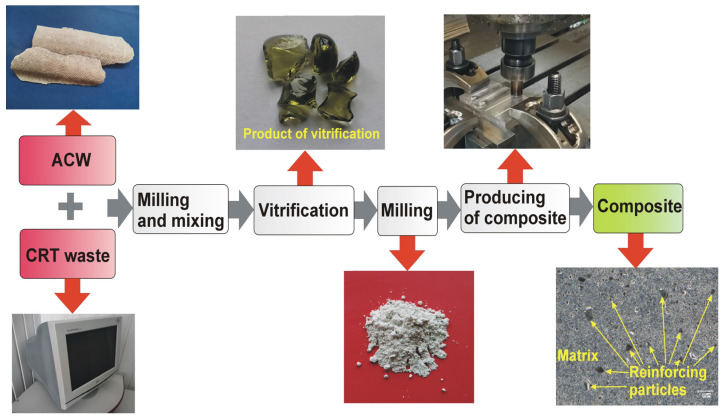
Scheme for producing composite.

As part of this work, an attempt was made to use the product of vitrification of ACW and CRT cullet as a reinforcing phase in the production of metal-matrix surface composites. The composite reinforced with the vitrified material was prepared according to the scheme shown in Figure 12. The work highlights both the application potential of the product resulting from the processing of ACW and glass from CRTs, as well as the advantages of the employed disposal technology. The issue of utilising waste or its recycling/utilisation products to produce new products, and thus limiting the environmental effects resulting from storing this waste in landfills, is particularly important in the case of materials whose impact on the environment is clearly negative.

The research conducted as part of this work demonstrated that vitrification is a solution that enables the effective processing of two different wastes into a completely non-toxic material that can be used as a reinforcing phase in a metal-matrix composite. The vitrification process also eliminated the fibrous morphology of asbestos fibres, which is the main cause of its pathogenic properties. Beneficial microstructural effects were found in the composite produced using the FSP method, resulting in an improvement in the key performance properties of the AA7075 aluminium alloy. The formation of a composite microstructure, strong grain refinement, and an increase in the hardness and wear resistance of the material are the main effects that were found after FSP. The enhancement of the mechanical and tribological properties is a consequence of the introduction of hard particles into the composite matrix, but also of high grain refinement. The significant grain refinement observed in friction-stir-processed samples is primarily due to dynamic recrystallization, the main mechanism responsible for grain refinement in these materials. It is worth noting, however, that the final properties of the composites are a result not only of the processes occurring during FSP, but also of the processes occurring during the formation of the reinforcing phase, i.e., the vitrified material. These properties are, of course, influenced by the material’s structure and the chemical composition of the components used to produce the vitrified material. The presence of hard oxides derived from glass cullet (SiO_2_, Al_2_O_3_, ZrO_2_) or asbestos-cement waste (SiO_2_, Al_2_O_3_) shaped the properties of the vitrified material and simultaneously contributed to the formation of the composite’s properties. Magnesium oxide, present in ACW, also significantly affects the properties of the vitrified material. This oxide enhances the chemical stability and mechanical durability of the glass, contributing to the increased hardness and wear resistance of the final composite.

It is worth emphasising that the properties of composites reinforced with vitrified material were similar to the properties of an analogous composite, but reinforced with silicon carbide particles, which proves the potential of the used waste materials. One of the key conditions for composites that determines their proper and effective operation is good bonding of the reinforcing phase with the matrix. The performed tests found that the particles of the vitrified material were well bound in the matrix melt, and there was no particle agglomeration effect in the composite matrix, which proves the correct selection of processing parameters.

Based on the conducted research and the obtained results, it can be concluded that the use of vitrification products in the production of composites is fully justified and is an effective form of management of the above-mentioned waste products. This makes it possible, for example, to replace more expensive engineering materials such as SiC, Al_2_O_3_, Cr_2_O_3_, BN powders, nanotubes, and others that are currently applied to strengthen composites. The use of the vitrification product of ACW and CRT cullet as a reinforcing phase in composites leads to a reduction in the total costs of composite production and may ultimately contribute to reducing the amount of the above-mentioned waste stored in landfills and reducing the associated environmental costs. It can also be a factor stimulating the development and expansion of this waste disposal technology.

Taking into account the properties of composites reinforced with vitrified material, these composites could find applications similar to those of aluminium matrix composites reinforced with SiC particles. The automotive industry could be a major recipient of such composites. Competitive composites reinforced with SiC particles are used, among other things, in the production of engine parts and braking system components. Composites reinforced with vitrified material particles could also find applications in the mechanical engineering, aerospace, and transportation industries, as well as in other applications requiring abrasion resistance while maintaining core ductility and low product weight.

## Figures and Tables

**Table 1:** Elemental content in AA7075 alloy [Result of own research].

Alloy	Element Content (wt%)								
AA7075	Al	Zn	Mg	Cu	Fe	Si	Cr	Mn	Ti
	89.55	5.5	2.4	1.6	0.3	0.2	0.2	0.15	0.1

**Table 2:** Results of the analysis of the chemical composition of ACW.

Sample	Content, wt%							
ACW	CaO	SiO_2_	Fe_2_O_3_	MgO	Al_2_O_3_	SO_3_	K_2_O	rest
	69.92	14.80	4.44	4.15	2.47	2.57	0.27	1.38

**Table 3:** Chemical composition of CRT glass cullet.

Sample	Content, wt%							
CRT glass cullet	SiO_2_	BaO	SrO	Na_2_O	K_2_O	Al_2_O_3_	ZrO_2_	rest
	60.49	8.37	7.77	9.49	6.24	3.05	2.57	2.02

**Table 4:** Chemical composition of vitrification product.

Sample	Content, wt%					
Vitrified material	SiO_2_	CaO	SrO	BaO	ZrO_2_	MgO
	29.5	34.01	11.09	5.86	3.88	1.59
	Fe_2_O_3_	Al_2_O_3_	Na_2_O	K_2_O	rest	
	2.31	3.91	1.80	3.37	2.66	

**Table 5:** Mean hardness values and confidence intervals.

Test Interval [mm]	Mean Value		Confidence Interval +/−	
	AA7075/ Vitrified Material	AA7075/ SiC	AA7075/ Vitrified Material	AA7075/ SiC
0–1 mm	138.8	142.1	4.8	4.2
1–2 mm	139.5	142.5	2.4	5.1
2–3 mm	131.3	143.2	5.2	2.8
3–4 mm	130.7	136.3	4.5	4.5
4–5 mm	114.5	121.0	8.4	13.0
0–5 mm	134.9	140.5	5.9	5.0

## Data Availability

The original contributions presented in this study are included in the article. Further inquiries can be directed to the corresponding author.
